# Journaling as a Tool for Care Partners to Process and Stay Engaged Between Intervention Sessions

**DOI:** 10.1093/geroni/igaf122.675

**Published:** 2025-12-31

**Authors:** Jacqueline Eaton, Sarah Neller, Moroni Fernandez Cajavilca, Julene Johnson, Lee Ellington

**Affiliations:** University of Utah, Salt Lake City, Utah, United States; University of Tennessee, Knoxville, Tennessee, United States; University of Utah, Salt Lake City, Utah, United States; University of California, San Francisco, San Francisco, California, United States; University of Utah, Salt Lake City, Utah, United States

## Abstract

The Enhancing Active Caregiver Training (EnACT) intervention was developed to improve how care partners of persons living with dementia (PLWD) interact during training and support groups. During the pilot randomized trial, three sessions of EnACT were delivered every two weeks to 30 care partners of PLWD. To enhance retention and engagement during the two-week interval between intervention sessions, we provided optional take-home exercises. Each exercise included two open-ended questions: one related to the previous session (e.g., exploring relationship changes; what it means to keep going) and another free-write on a topic of choice. Response rates were high with 86% of participants completing all take-home exercises; the average completion time was 13 minutes per exercise. We conducted an inductive thematic analysis of the journal entries. Two major themes were identified: 1) New Connections Through Shared Caregiving Roles and 2) Persistence through challenges. Participants experienced loneliness due to caregiving demands but found friendship through shared experiences. They faced challenges related to illness, behavioral symptoms, financial concerns, changes in temperament, and loss. Perseverance was essential; faith, gratitude, and moments of connection helped them “keep going.” The take-home exercises encouraged participants to reflect and expand upon EnACT topics between sessions, likely contributing to high retention rates. Post intervention participants rated exercises positively and appreciated the opportunity to “sit down and think” and “formulating your thoughts was helpful.” Optional take-home exercises reinforced EnACT and supported retention. Future efforts will explore journaling as a tool to enhance application of EnACT beyond the intervention space.

